# Comparing the self-reported health-related quality of life (HRQoL) of artisanal and small-scale gold miners and the urban population in Zimbabwe using the EuroQol (EQ-5D-3L+C) questionnaire: a cross-sectional study

**DOI:** 10.1186/s12955-020-01475-0

**Published:** 2020-07-29

**Authors:** Jana Becker, Stephan Bose-O’Reilly, Dennis Shoko, Josephine Singo, Nadine Steckling-Muschack

**Affiliations:** 1 Institute and Clinic for Occupational, Social and Environmental Medicine, University Hospital, LMU Munich, 80336 Munich, Germany; 2 Institute for Medical Information Sciences, Biometrics, and Epidemiology - IBE, LMU Munich, 81377 Munich, Germany; 3 Institute of Health Economics and Health Care Management, Helmholtz Zentrum München GmbH, German Research Center for Environmental Health, 85764 Neuherberg, Germany; 4 Institute of Public Health, Medical Decision Making and Health Technology Assessment, UMIT Private University for Health Sciences, Medical Informatics and Technology, Eduard Wallnoefer Center I, 6060 Hall in Tirol, Austria; 5 Tailjet Consultancy Services, Vainona, Borrowdale, Harare, Zimbabwe; 6 National University of Science and Technology, PO Box AC 939, Ascot, Bulawayo, Zimbabwe

**Keywords:** Health-related quality of life (HRQoL), EuroQol (EQ-5D + C-3 L), Artisanal and small-scale gold mining (ASGM), Mercury, Mercury intoxication, Minamata convention on mercury

## Abstract

**Background:**

The role of artisanal and small-scale gold mining (ASGM) as a source of income is rapidly gaining importance in the economically difficult times in Zimbabwe. Besides limited epidemiological data, no data about the self-reported health-related quality of life (HRQoL) of artisanal and small-scale gold miners exist. The aim of the project was to access HRQoL of ASGM workers to improve the data base and compare the data to the urban Zimbabwean population.

**Methods:**

Data from 83 artisanal and small-scale gold miners in Kadoma, Zimbabwe was analysed. The HRQoL was assessed using the EuroQol dimensions (mobility, self-care, usual activities, pain/discomfort, anxiety/depression) accompanied by the cognition add-on questionnaire (EQ-5D-3L+C) and associated visual analogue scale (VAS). We described the EQ-5D dimensions and VAS values and computed health utility (HU) values using the Zimbabwean tariff. The proportions of miners reporting no problem in each EQ-5D dimension were compared with corresponding proportions reporting any problem (moderate or severe), and mean HU and VAS values were analysed across subgroups of the sample. To test differences between subgroups, Fisher’s exact test was used and between urban and mining population, Student’s t-test was used.

**Results:**

The reported health states of miners were homogenous, with a large amount (42%) reporting ‘full health’. Mean (SD) VAS and HU values were 81.0 (17.5) and 0.896 (0.13), respectively. Subgroup analysis showed that miners with a lower education reported significantly more problems in the dimension of daily activities and miners with mercury contact had more problems in the dimensions of pain/discomfort and cognition. Comparison between mining and urban population showed that in the oldest age group, self-rated VAS values of miners were significantly higher than of their urban counterparts.

**Conclusions:**

There were no significant differences in the HRQoL of mining and urban populations. However, the reason might be adverse health effects faced by the urban population that do not apply to rural mining areas. A higher education level of miners can improve the HRQoL, which is especially impaired by problems in the cognition dimension.

## Background

Artisanal and small-scale mining (ASM) is characterised by complex interactions of environmental, economic, technological, social, and health factors. Most definitions include one or more of the following aspects: labour-intensive work, limited mechanisation, low productivity and capital, limited access to land and markets, an informal workforce, and deposit exploitation/sterilisation [1–5].

In Africa, ASM occurs in almost all countries, where more than 10 million people are directly engaged in this sector. However, as ASM is informal or illegal in its nature and is characterised by geographical and seasonal shifts, exact numbers are difficult to determine. Nonetheless, it can be stated with certainty that poverty and high commodity prices lead to the ASM growth trends [6]. This especially applies to artisanal and small-scale gold mining (ASGM) [7].

In Zimbabwe, ASGM plays a significant role in the country’s economy, both in terms of employment figures and national gold output, accounting for more than half of the output and with more than 500,000 people being directly engaged in the sector, and several million dependents [8, 9]. High unemployment and lack of other employment options further increase the importance of the sector. In the fourth quarter of 2016, artisanal and small-scale miners produced more gold than large-scale mining companies and are in control of up to 65% of the current gold deposits in the country [10].

### Occupational health of artisanal and small-scale miners

Gold mining processes by ASGM in Zimbabwe today largely rely on technologies introduced over 100 years ago and which involve stamp mills, amalgamation plates and cyanidation circuits. There are two main reasons that have led to the preservation of those trusted and well-established methods: the fact that most of the mining and processing technology is simple, rapid and transparent while the few known alternatives have not been adequately demonstrated. Unfortunately, for the introduction of cleaner technologies, miners are not worried about negative health or environmental negative impacts. The inefficiency of the old and tried methods as well as pollution aspects are not important for the miners. As a result, efforts at introducing new and cleaner technologies have been relatively unsuccessful [11].

Outdated and polluting technologies together with unsafe working conditions, a lack of personal protective equipment and security measures, lead to a large number of health and environmental hazards related to gold mining, ASGM in particular. Along with the increasing importance of the ASGM sector in Zimbabwe, with a growing number of miners, the role of occupational health becomes ever more significant for the overall health of the Zimbabwean population [12].

The main health hazards for miners are noise, injuries, silica dust, and mercury which occur at different stages of the mining process. ASGM is further related to specific community health hazards that affect miners and their families, including crowded living conditions and stress [13]. Other social deteminants of health that effect miners are lack of infrastructure, unsafe and poor living conditions and migration. The latter is very prevalent in many African countries, as artisanal and small-scale miners are highly mobile, which leads to a disruption of traditional familiy structures with many correlated health hazards [13–15]. Nearly all of the presented health hazards have already been documented in Zimbabwe as well [16–19].

The extensive and ongoing use of mercury in ASGM is an especially serious problem. On a global scale, ASGM is responsible for approximately 37% of all mercury emissions and is the largest source of air and water mercury pollution. The African region is estimated to have the highest population impacted by mercury pollution worldwide [20]. Mercury is used in the extraction process of gold where its high affinity for gold helps to catch the gold from ore pulp and the resultant amalgam is eventually burnt to release the mercury while freeing up the gold. During the process mercury vapour is released into the atmosphere [21, 22] that almost always exceeds the WHO’s limit for public exposure of 1.0 μg/m^3^ [23]. Mercury vapour can travel long distances before being deposited into waterways and soils [24]. The dimension of the effect of mercury exposure in ASGM areas was quantified by a burden of disease study in Zimbabwe and is within the top 20 health hazards. The study established that up to 72% of miners are affected by chronic mercury intoxication [25].

Miners in ASGM often live close to the mining sites in small villages. Therefore, environmental pollution not only affects the miners, but their whole families [14]. They work in small groups and are often paid in shares of the gold [9]. ASGM in Zimbabwe is mainly licensed (approximately 70%) or informal mining (approximately 30%), but around 70% of the miners are unskilled [9].

### Influence of occupational hazards on health-related quality of life

The possible effects of exposure to all occupational hazards can have a negative impact on the health of miners, including mercury intoxication which is linked to commonly known symptoms like kidney dysfunction, autoimmune disease, and neurological symptoms [26]; while the exposure to dust can cause silicosis [27]; uncorrected hearing loss due to high levels of noise can lead to isolation, reduced social activity, and symptoms of depression [28]. Additionally, potential long-term effects related to ASGM are tuberculosis, chronic obstructive pulmonary disease, and chronic bronchitis. Furthermore, high workload, repetitive tasks, low effort-reward imbalance and the unsafe working environment can lead to stress and mental health problems [29].

When taking into account the massive amount of health risks related to ASGM, we assumed that being a miner has a significant effect on health-related quality of life (HRQoL) of miners. This effect of mining will increase in importance with growing numbers of miners and the assumption that this trend will continue for the foreseeable future [9].

### Aim of study

Even though ASGM is a widespread phenomenon around the globe, there is no unique solution to solve the common problems and meet the health needs of artisanal and small-scale gold miners. As regional, historical, cultural and economic forces lead to highly diverse socio-economic environments and social attitudes, structures are created that limit the communities’ abilities to develop and adopt more efficient and less polluting mining practices [11]. Therefore, a better understanding of the general health and well-being of artisanal and small-scale miners, their HRQoL and the different types of hazards in the mining environment may lead to new opportunities for more successful and appropriate occupational health interventions [29].

The aim of this study was to assess self-reported HRQoL of ASGM workers in Zimbabwe, to calculate health utility (HU) values and compare the results with findings from the urban Zimbabwean population. This study will enable further comparisons of miners’ HRQoL between different mining regions, other professions or parts of the population and provide information to guide further decision-making regarding health-improving interventions.

The hypothesis was that, since artisanal and small-scale miners suffer from more limitations to health than the urban population due to the exposure to occupational hazards, they would report a lower self-reported HRQoL. Further hypotheses were that different aspects additionally influence the miners’ health, including age, a low education level, period of time working as a miner and contact with mercury were assumed to have a negative effect on HRQoL. An aspect we also wanted to explore was the difference of HRQoL between men and women.

The content of this paper assists Zimbabwe in fulfilling the requirements of the Minamata Convention on Mercury through the gathering of health data and is in line with the Sustainable Development Goals, especially the third goal ‘Mining, Good Health and Well-being’ [30, 31].

## Methods

This is an epidemiological, observational study with a cross-sectional design. Data was collected in a two-week field study in Kadoma District, Zimbabwe. Kadoma District is located approximately 150 km west of Harare, the capital of Zimbabwe.

### Procedure

The target population consists of all artisanal and small-scale miners in Kadoma district. Estimations conclude that of a total population of approximately 310,000 inhabitants 50% are directly or indirectly involved in mining and milling activities. More specific, roughly 30,000 miners are estimated to live in the region with 120,000 dependent family members [24, 32]. The target population has been limited to these approximately 30,000 miners. All participants that identified themselves as small-scale miners were allowed in the sample.

To contact the target population, snowball sampling was used. Local partners that already worked with the miners before made the initial contact with relevant miners at different mining sites. Those then arranged the contact to other miners. Snowball sampling was used because the miners were difficult to contact, being reluctant to be questioned in the beginning. Starting with community leaders at different mining sites led to trust in the population and was the basis to conduct further inquiries [33]. The participants were questioned in English and in person by the same researcher, with the same translator always being on side in case they were needed. Standardised and before data collection rehearsed explanations were given if questions were unclear to the participant.

### Measures

The influence of the occupational hazards caused by working in artisanal and small-scale gold mines on the miners’ lives was investigated by analysing their HRQoL with help of the five EuroQol dimensions together with the cognitive add-on (EQ-5D-3L+C) questionnaire. The questionnaire included five dimensions of health: mobility, self-care, usual activities, pain and discomfort, and anxiety and depression [34]. The EuroQol Research Foundation gave their permission to the questionnaire use. In this study the additional dimension cognition (C) was added, as was done previously by Stouthard et al. (1997) [35], resulting in an overall number of 729 possible health states, because impairment of cognitive abilities is one of the possible consequences of mercury intoxication. A further study exploring the effect of adding an additional dimension concluded that by including a cognitive attribute the concept of health becomes more comprehensive [36].

The 3 L version contains three levels of possible answers for each dimension: no problems (1), moderate problems (2), severe problems (3). From the respective answers a health state of HRQoL can be computed, representing the individual’s overall health. The best imaginable health state is thus represented by ‘111111’. Further, each respondent was asked to value his or her own health status on the EQ-5D visual analogue scale (VAS). The scale ranges from zero, which represents the ‘worst possible health state’ the respondent can imagine, to the ‘best possible health state’ at 100 [34].

The EQ-5D questionnaire is applicable to a wide range of health conditions and treatments and can be used in population health surveys, and economic and clinical evaluation of health care. The main advantages are cognitively undemanding questions and the short time it takes to complete the questionnaire [34]. We used the English version of the generic EQ-5D-3L system, which was confirmed to be appropriate for the Zimbabwean population by Jelsma et al. [37].

An additional questionnaire was used to conduct the following covariates: age, gender (female/male), highest education (primary, secondary, post-school), contact with mercury (yes/no) and years in mining (less than five/more than five).

### Statistical analysis

To answer the research question, four main analyses were performed: first, we described the HRQoL of miners working in the ASGM sector regarding the EQ-5D items and the additional dimension of cognition. Also, the self-rated VAS of miners was illustrated.

Second, we computed health utility (HU) values. HUs were generated based on the dimensions of the EQ-5D to illustrate the individuals’ health status, weighted by an appropriate population. In this study we used the Zimbabwean tariff that was developed with the time trade-off method by Jelsma et al. [37]. As no values exist for the cognition dimension, HUs could only be calculated for the regular dimensions of the EQ-5D. They range from zero to one, with zero representing ‘death’ and one representing ‘full health’ [34]. Student’s t-test was used to examine differences in mean values between the value sets and VAS. For this test VAS values were divided by 100 to make comparisons possible [38].

Third, for sub-group analyses we dichotomized the population of ASGM workers into miners without a problem and miners with a problem (moderare probleme and severe problem), then examined the respective proportions for each dimension. Further, we calculated mean HU and VAS values. The procedure was applied for the total population of miners and across the specific characteristics according to the covariates. Fisher’s exact test was used to test the differences between the subgroups in univariate analysis, due to the small sample size.

Finally, to compare the VAS and HU values of the sample with the urban Zimbabwean population, we used the previously published reference values of 2384 randomly selected residents of Glenview, a high-density suburb of Harare. Persons in that sample had to be at least 15 years old to be included in the study, making the population comparable to our sample [37]. The sample from Glenview provides the only available VAS and HU data from Zimbabwe before the current study. To examine differences between the samples, again Student’s t-test was used.

All analyses were conducted with a significance level of 95% and using IBM SPSS Statistics, Version 25.

## Results

A total of 83 artisanal and small-scale gold miners working in the area of Kadoma District were questioned of which 83.1% were male (Online Supplementary Table 1). The age of the miners ranged from 19 to 70 with a mean age (SD) of 35.3 years (10.3). In comparison with population statistics, males in the age between 25 and 44 were overrepresented in the study. However, this distribution of the sample is consistent with the characteristics of the Zimbabwean small-scale mining work force [9]. Compared to the population estimate (44%), the sample was better educated with 73.5% indicating they finished secondary school which accounts for 13 years of schooling.

The income of miners varies greatly from month to month, as most miners are paid as a proportion of produce or in shares. Therefore, many could only give rough estimates of their income. With nearly 50%, the vast majority has an income between 100$ and 500$, while 12% indicated an income less than 100$. An income between 501$ and 1000$ was reported for 18% while the last 16% earn more than 1000$. In comparison, the gross domestic product (GDP) per capita in Zimbabwe was at 998$ in 2016 [39].

The work experience of the miners in the sample differs significantly, ranging from 2 weeks to 40 years. However, the average work experience is 7.5 years with a median of 5 years.

### EQ-5D-3L+C health states of miners

All participants answered to all six dimensions of HRQoL addressed in this study. Among the 729 possible health states defined by the EQ-5D-3L system in combination with the additional dimension cognition, a total of only 27 health states were identified in the sample With 42% at ‘full health’ (111111), it was easily the most common health status. Of those 27 health states, the 10 most common ones include about 80% of the sample. None of these contains a level of extreme severity. The health status with the highest dimensions reported was 212,322, relating to a single miner.

**Fig. 1:**
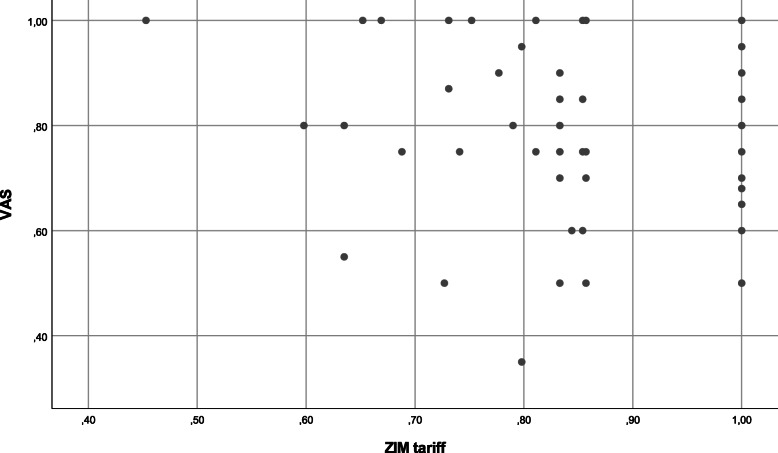
Scatterplot of self-perceived VAS values and HUs calculated with ZIM tariff

Of the total population of miners, 67.5% reported no problem, or just one moderate problem in one dimension. In contrast, 32.5% reported problems in more than one dimension or at least one severe impairment. In total, 13.3% of miners reported severe problems in at least one dimension. A list of all reported health states and a figure giving an overview about the most common health states can be found in the Online Supplementary (Online Supplementary Table *2*, Figure 1).

Table 1 presents the distribution of individuals by severity level for each EQ-5D-3L+C dimension in total and by age group. In all six dimensions the most frequently reported response was ‘no problem’: mobility (88%), self-care (92.8%), usual activities (88%), pain/discomfort (78.3%), anxiety/depression (75.9%) and cognition (71.1%).

**Table 1:** Self-reported health state of miners in mining areas in Kadoma (*n* = 83) and Fisher’s exact test for age group differences. Frequency of levels in each dimension are presented as percentages

EQ-5D DIMENSIONS		AGE GROUPS				***p***-value	Total
		15–24	25–34	35–44	45+		
**Mobility**	No Problems	100	87.8	81.0	92.3		**88.0**
	Problems	0.0	12.2	19.0	7.7	0.673	**12.0**
	Severe Problems	0.0	0.0	0.0	0.0		**0.0**
**Self-Care**	No Problems	75.0	92.7	95.2	100		**92.8**
	Problems	12.5	7.3	4.8	0.0	0.212	**6.0**
	Severe Problems	12.5	0.0	0.0	0.0		**1.2**
**Usual Activities**	No Problems	75.0	87.8	85.7	100		**88.0**
	Problems	25.0	12.2	14.3	0.0	0.329	**12.0**
	Severe Problems	0.0	0.0	0.0	0.0		**0.0**
**Pain** **Discomfort**	No Problems	87.5	85.4	71.4	61.5		**78.3**
	Problems	12.5	12.2	28.6	30.8	0.309	**19.3**
	Severe Problems	0.0	2.4	0.0	7.7		**2.4**
**Anxiety** **Depression**	No Problems	87.5	78.0	71.4	69.2		**75.9**
	Problems	12.5	17.1	28.6	23.1	0.792	**20.5**
	Severe Problems	0.0	4.9	0.0	7.7		**3.6**
**Cognition**	No Problems	62.5	72.2	71.4	69.3		**71.1**
	Problems	25.0	19.5	19.0	30.8	0.878	**21.7**
	Severe Problems	12.5	7.3	9.5	0.0		**7.2**

In more detail, differences can be identified between the age groups. Younger miners report more problems in the dimensions self-care and usual activities, and older miners in the dimensions pain/discomfort and anxiety/depression. While problems with mobility are rare in all age groups, problems with cognition are often reported in all of them, with very low difference between the groups (*p* = 0.673). In the overall sample the highest percentage of moderate or severe problems in any dimension was reported in the cognition dimension (28.9%). None of the differences observed between age groups were statistically significant.

### VAS and HU values of miners

The mean VAS value was 81.0 (17.5), with a maximum of 100 and a minimum of 35. In this sample, the self-reported mean quality of life (QoL) by miners in Kadoma can therefore be described with a value of 0.81 **(**Table 2**)**. When applying the Zimbabwean tariff, a mean HU of 0.896 was calculated, which was significantly higher than the self-rated HRQoL of miners.

**Table 2:** Paired Student’s t-test comparison of self-rated VAS values by miners in Kadoma with HU values based on the Zimbabwean tariff

	Mean (SD)	***p***-value
**Self-rated QoL (VAS)**	0.810 (0.17)	< 0.001*
**HU ZIM-Tariff (TTO)**	0.896 (0.13)	

*p < 0.05

However, when looking at the results in more detail (), quite a few miners with low HU values, which is synonymous with more problems in the dimensions, have reported VAS values of 100 resembling ‘perfect health’.

### Differences between subgroups

A detailed analysis of the subgroups **(**Table 3**)** showed that men reported less problems in the dimensions mobility (*p* = 0.060), usual activities (*p* = 0.674), and pain/discomfort (*p* = 0.491), while they experience more impairments in the dimensions self-care (*p* = 1.000), anxiety/depression (*p* = 1.000), and cognition (*p* = 1.000). However, these differences are small and none of them is statistically significant. Even though the amount of female miners reporting no problems (29%) is lower than of males (45%), the HU values are almost identical (*p* = 0.188). In contrast, the mean self-rated VAS value of women was higher than of men (*p* = 0.494).

**Table 3:** Univariate subgroup comparisons in the sample of artisanal and small-scale miners (*n* = 83) using Fisher’s exact test. Frequencies of reported problems are reported as absolute numbers and percentages

	N (%)	No problem N (%)	Problem with mobility N (%)	Problem with self-care N (%)	Problem with daily activities N (%)	Problem with pain/ discomfort N (%)	Problem with anxiety/ depression N (%)	Problem with cognition N (%)	Mean health utility (SD) ZIM tariff	Mean VAS (SD)
**Total**	83 (100)	35 (42.2)	10 (12.0)	6 (7.2)	10 (12.0)	18 (21.7)	20 (24.1)	24 (28.9)	0.896 (0.13)	81.0 (17.5)
**Sex**		*p* = 0.374	*p* = 0.060	*p* = 1.000	*p* = 0.674	*p* = 0.491	*p* = 1.000	*p* = 1.000	*p* = 0.188	*p* = 0.494
Men	69 (83.1)	31 (44.9)	6 (8.7)	5 (7.2)	8 (11.6)	14 (20.3)	17 (24.6)	20 (29.0)	0.898 (0.13)	80.0 (18.0)
Women	14 (16.9)	4 (28.6)	4 (28.6)	1 (7.1)	2 (14.3)	4 (28.6)	3 (21.4)	4 (28.6)	0.885 (0.12)	86.1 (14.7)
**Age**		*p* = 0.400	*p* = 0.673	*p* = 0.249	*p* = 0.329	*p* = 0.228	*p* = 0.808	*p* = 0.917	*p* = 0.238	*p* = .0323
15–24	8 (9.6)	4 (50.0)	0 (0.0)	2 (25.0)	2 (25.0)	1 (12.5)	1 (12.5)	3 (37.5)	0.885 (0.13)	86.9 (17.1)
25–34	41 (49.4)	20 (48.8)	5 (12.2)	3 (7.3)	5 (12.2)	6 (14.6)	9 (22.0)	11 (26.8)	0.908 (0.13)	81.2 (19.4)
35–44	21 (25.3)	8 (31.1)	4 (19.0)	1 (4.8)	3 (4.3)	6 (28.6)	6 (28.6)	6 (28.6)	0.894 (0.11)	77.0 (14.2)
44+	13 (15.7)	3 (23.1)	1 (7.7)	0 (0.0)	0 (0.0)	5 (38.5)	4 (30.8)	4 (30.5)	0.866 (0.13)	83.3 (16.0)
**Education**		*p* = 0.123	*p* = 0.499	*p* = 0.767	***p*** = 0.035*	*p* = 0.902	*p* = 0.061	*p* = 0.066	*p* = 0.178	*p* = 0.073
Primary	21 (25.3)	5 (23.8)	4 (19.0)	2 (9.5)	6 (28.6)	5 (23.8)	9 (42.9)	10 (47.6)	0.844 (0.14)	87.38 (17.4)
Secondary	57 (68.7)	27 (47.4)	6 (10.5)	4 (7.0)	4 (7.0)	12 (21.1)	10 (17.5)	14 (24.6)	0.911 (0.12)	78.86 (17.6)
Post-school	5 (6.0)	3 (60.0)	0 (0.0)	0 (0.0)	0 (0.0)	1 (20.0)	1 (20.0)	0 (0.0)	0.937 (0.09)	79.00 (9.6)
**Years in Mining**		*p* = 1.000	*p* = 0.748	*p* = 0.683	*p* = 0.748	*p* = 0.426	*p* = 0.441	*p* = 0.808	*p* = 0.331	*p* = 0.065
< = 5 Jahre	45 (54.2)	16 (42.1)	6 (13.3)	4 (8.9)	6 (13.3)	8 (17.8)	9 (20.0)	14 (31.1)	0.897 (0.13)	81.78 (17.9)
> 5 Jahre	38 (45.8)	19 (42.2)	4 (10.5)	2 (5.3)	4 (10.5)	10 (26.3)	11 (28.9)	10 (26.3)	0.894 (0.12)	80.13 (17.2)
**Contact with Mercury**		*p* = 0.203	*p* = 0.697	*p* = 1.000	*p* = 1.000	*p* = 0.540	*p* = 1.000	*p* = 0.781	*p* = 0.405	*p* = 0.752
No	20 (24.1)	11 (55.0)	3 (15.0)	1 (5.0)	2 (10.0)	3 (15.0)	5 (25.0)	5 (25.0)	0.915 (0.12)	76.90 (19.9)
Yes	63 (75.9)	24 (38.1)	77 (11.1)	5 (7.9)	8 (12.7)	15 (23.8)	15 (23.8)	19 (30.2)	0.890 (0.13)	82.33 (16.6)

* *p* < 0.05

In almost all dimension a higher education is consistent with less problems. While 60% of miners who attended more than 13 years of school and 47.4% of miners who finished high school report no problems in any dimension, the percentage in the subgroup of miners who just finished primary school (23.8%) is much lower (*p* = 0.123). Only for the dimension of daily activities this effect was significant. In coherence with the findings for each dimension, the HU value is higher for miners with a better education (*p* = 0.178). However, the self-reported VAS value is lower in the highest educated group in comparison to the lowest educated group (*p* = 0.073).

Regarding all examined aspects, the years in mining do not seem to have an effect. Differences between the groups are small in all dimensions, as well as HU and VAS values, and are not significant, nor consistent in their direction of the effect.

A total of 63 miners had contact with mercury while working as miners. Of those 20 who had never worked with mercury before, 55% reported no problems in any dimension, while the proportion in the group that had contact with mercury is a bit lower (38%) (*p* = 0.203). In the group of miners with mercury contact, a higher amount of problems can be seen, especially in the dimensions cognition and pain/discomfort, but also in the dimensions self-care and daily activities. In the other two dimensions the miners with no mercury contact report more problems. The differences between the two subgroups regarding the dimensions were all not significant. HU values are slightly lower in the group with mercury contact, but VAS values are higher. Therefore, we controlled for a confounding effect of income, of being a mine owner, and of working longer than 5 years as a miner, but none of these characteristics were significantly differently distributed between the groups. The reported health states of miners with mercury contact are presented in the Online Supplementary (Online Supplementary Table *3*). These health states are very similar to those of the overall population. Except for the one significant difference mentioned before, none of the disparities identified between the subgroups were statistically significant.

### Comparison of miners’ HRQoL with urban population of Harare

When comparing the EQ-5D dimensions of miners in Kadoma with the urban population in Harare, miners reported more problems in the dimensions mobility, self-care and usual activities, while the urban population had more impairments in the dimensions pain/discomfort and anxiety/depression **(**Table 4**)**. The biggest difference can be seen in the dimension of pain/discomfort, where the urban population reports a higher amount of impairments, but overall differences are small.

**Table 4:** Self-reported health status of miners in Kadoma (*n* = 83) compared to subjects in urban Zimbabwe (*n* = 2183). Frequencies of levels in each dimension are reported as percentages

Dimension	Mobility		Self-Care		Usual Activities		Pain/ Discomfort		Anxiety/ Depression	
	Miner	City^a^	Miner	City^a^	Miner	City^a^	Miner	City^a^	Miner	City^a^
**No Problems**	88.0	90.1	92.8	96.5	88.0	89.0	78.3	69.5	75.9	69.3
**Problems**	12.0	9.7	6.0	3.4	12.0	10.5	19.3	26.3	20.5	23.9
**Severe Problems**	0.0	0.1	1.2	0.1	0.0	0.6	2.4	4.2	3.6	6.8

^a^ Compared to data from Jelsma et al. [37]

The comparison of self-reported mean VAS values showed **(**Table 5**)** that the urban population reported lower values in women and in all age groups, with the only exception being the urban male population, as they reported a slightly higher mean VAS value in contrast to the miners. Only the difference in the highest age group was significant.

**Table 5:** Unpaired Student’s t-test comparison of VAS values of miners to the urban population of Zimbabwe

	Visual analogue scale			
	Miners (SD)	City (SD)	Difference Miners - City	***p***-value
**Total**	81.0 (17.5)	79.8 (19.4)	1,2	0.526
**Sex**				
Men	80.0 (18.0)	81.5 (18.0)	−1.5	0.490
Women	86.1 (14.7)	78.7 (15.2)	7.4	0.083
**Age group**				
18–24	86.9 (17.1)	81.8 (16.)	5.1	0.449
25–34	81.2 (19.4)	79.8 (16.6)	1.4	0.642
35–44	77.0 (14.2)	76.6 (19.5)	0.4	0.898
> 44	83.3 (16.0)	69.0 (34.7)	14.3	0.007*

* *p* < 0,05 * Compared to data from Szende et al. [40] & Jelsma et al. [37]

## Discussion

Addressing the research question, the self-reported HRQoL of artisanal and small-scale miners in Kadoma, Zimbabwe was surprisingly homogenous and a high number of miners (42%) have reported the best possible health state of ‘111111’. This result is consistent with results from Goa, India, where 43% of people living in mining regions reported no health problems [41].

### Main findings

The first main finding of our study is that, in contrast to our hypothesis, the self-reported HRQoL of artisanal and small-scale gold miners in Kadoma was not significantly lower than that of the urban Zimbabwean population, despite the high amount of health hazards miners are exposed to every day. Considering each dimension on its own, it becomes clear that the amount of reported problems is in relative equal balance, as only small differences could be identified. The fact that urban citizens report more problems in the dimension of pain/discomfort is surprising, as we assumed just the opposite. Also, reported VAS values were higher in all age groups, in the oldest age group the value was even significantly higher in the group of miners than for their urban counterparts. This is even more relevant as the VAS value in the highest age group, in contrast to our hypothesis, was also higher than that of the younger miners, despite age having a documented independent negative effect on HRQoL [42].

The reasons for these circumstances are complex, and we identified several causes: first, a similar observation was made by Amponsah-Tawiah et al. [29] who examined the quality of life of miners in the Ghanaian mining industry. They concluded that older employees were given less stressful jobs, had a more regular work pattern and lighter schedules, leading to a better health and well-being of older miners in comparison to younger ones. However, these miners were working in large-scale mines, which are expected to be more organised than small-scale mines [29]. That a similar finding could be documented in a less organised setting like small-scale mining as well leads us to another conclusion: we assume the healthy worker effect is one of the main reasons, leading to higher VAS values in older miners, as well as in higher VAS values in miners in comparison to the urban population. Only the healthiest miners can continue working into old age [43].

Another reason could be the declining Zimbabwean economy: Since 1998 formal employment options in Zimbabwe have been decreasing, leading to a situation where less than 20% of the population were in formal employment in 2004, and more than 80% working in the informal sector. Up until now, the economic situation has not seen any improvement [39, 44]. In this context, most employment options in the informal sector are in agriculture and mining, to which the urban population has only limited access [9, 44]. This leads to a population that can be described as ‘the urban poor’, which are considered especially affected by the double burden of disease in the process of the ‘epidemiological transition’ [45].

Supporting this explanation, the income of miners seems to be lower than that of the general population at first sight only. A closer look shows that the distribution of gross domestic product (GDP) in Zimbabwe is very uneven, with a Gini coefficient of income of 43.20 in 2011 and roughly 50% of the income share held by the highest 20% of the population [46]. This demonstrates that the vast majority of the population has an income much lower than the GDP per capita, suggesting that the average income of miners is generally higher than in other professions and sectors of the economy. Considering the high unemployment levels in the economy, mining remains one of the few options for Zimbabweans to earn an income at all, resulting in the fact that the negative impact of occupational health hazards on HRQoL might be more than compensated by the positive effect of higher incomes [9].

Second, it is not surprising that when compared self-reported VAS values are lower than calculated HU values, since the latter are based on the Zimbabwean tariff which was calculated using the time trade-off (TTO) method [37]. The HRQoL is usually worse when the VAS method is used in comparison with the TTO model [47]. When looking at Fig. 1 these circumstances explain the fact that many miners reported lower VAS values, despite a calculated HU value of ‘1’.

However, there was also a considerable number of miners reporting VAS values of 100, in spite of low HU values and impairments in several dimensions. One explanation for this could be the *disability paradox*, which describes the phenomenon where people with a severe disability or illness report to have a good quality of life [48]. Even though being a miner is not ‘a disease’, we believe that here the same mechanisms could be at play, as Noronha & Nairy [41] observed a similar effect while analysing the QoL of people living in mining and non-mining villages. They found out that even though the overall living conditions in non-mining regions are significantly better, both groups valued these conditions with almost equal satisfaction levels. One explanation they gave is that people adjust to the living conditions and adapt their demands to lower standards [41].

In the context of the *disability paradox*, the following explanations were identified for reporting a high QoL despite impairments: contextual factors, the aspect of having control over themselves and possibility of participation [49, 50]. All of these aspects are different for miners in comparison to the aspect of disability. While disabled people are found to report a high QoL when they are able to maintain these factors despite disability, small-scale mining in Zimbabwe could support miners to additionally gain these abilities. By being able to earn an income, miners might achieve higher self-reliance, are better able to participate and more in control of contextual factors that could lead to higher reported HRQoL, even though miners experience physical and social impairments as well [14].

The third main finding is that in contrast to the overall population, miners seem to be better educated [32]. This is unexpected, as artisanal and small-scale miners usually work in informal or illegal environments, where no specific education is required. The high education of miners indicates that the overall education of the Zimbabwean population seems to be high, with the majority of Zimbabweans passing through secondary school. Further, this shows that even for highly educated Zimbabweans mining could be a good option to gain an income. Even though the relation between education levels and self-reported HRQoL is complex, Ross & Van Willingen [51] documented a correlation between higher education levels and better health states. Therefore, a higher education might be one of the reasons miners report less health problems. This finding is consistent with our hypothesis that a low education level has a negative impact on the health state of miners. Earlier findings of our study group showed that an increase in knowledge and education leads to a safer work environment, less accidents and a more responsible behaviour at work [14]. Even though the difference between the groups is only significant in the dimension of usual activities, the nature of the relationship is clear and consistent. In contrast, self-reported VAS values are lower for miners who are better educated. One explanation might be that miners with tertiary education prefer and take jobs other than mining whenever possible, as they are more aware of the occupational hazards and the negative impact of mining on their health. The fact that despite better health more educated people value their quality of life not significantly better than less educated ones is well documented, too [51].

### Further important findings

According to Steckling et al. [25] approximately 72% of ASGM workers in Zimbabwe in 2004 have some form of chronic mercury intoxication, we assumed that of the miners in our sample with mercury contact at least a few would show symptoms of chronic mercury intoxication as well. A circumstance we concluded would be visible in the reported health states, HU and VAS values. Even though differences between the groups of miners with or without mercury contact were not significant, we believe the higher amount of reported problems in the dimensions pain/discomfort and cognition could be an indicator for mercury intoxication [52]. Symptoms of chronic mercury intoxication are headaches, neuralgia or paraesthesia, which can be summarized under “pain” (Drasch). However, the results in the other dimensions and HU and VAS values do not support our hypothesis, of significantly impaired HRQoL due to contact with mercury.

Further, we compared the reported health states, to the health state of miners with mercury intoxication as expected by experts and published by Steckling et al. [53], who assumed health states for moderate or severe intoxication of ‘121222’ or ‘233333’. In our sample, not a single miner with mercury contact reported these exact health states (Online Supplementary Table *3*). Of the identified health states, four can be described as close to a moderate mercury intoxication and only one to a severe intoxication. In total, this is either contradicting the finding from the year 2004 that up to 72% of miners in Zimbabwe suffer from mercury intoxication, or the assessed HRQoL of intoxicated miners by experts has been overestimated. In accordance to our findings, especially the dimension of self-care, where in our sample 92% of miners with mercury contact reported no problems, seems to be regarded too low by Steckling et al. [53] for people with an intoxication. However, as we did not collect and analyse any human samples, it is not clear which of the questioned miners actually suffer from mercury intoxication. Again, the healthy worker effect could be relevant in this aspect and miners suffering from symptoms of mercury intoxication might not have been part of the sample. The prevalence of intoxicated miners, what was 72% in 2004, could have changed in the last 13 years.

Although the amount of women reporting no problems in any dimension is higher than the number of men, the minimal difference between the respective HU values of different genders demonstrated that valued by the Zimbabwean population there exist rarely any difference between the health states of men and women. In contrast, higher VAS values indicated that women in our sample despite reporting more problems, value their health status better than the men in the sample. The difference is not statistically significant, which can mainly be explained by the small number of women in the sample. Despite, one reason could be that women in Zimbabwe appreciate the opportunity to work and to be able to earn an income for themselves, which is often difficult for them in mining regions [14, 41]. Our results underline that an opportunity to work could influence the perception on health of women.

Regarding the negative effects of mining in the long-term perspective, we believe that the classification of less or more than 5-years was not optimal to for identifying the long-term effects of mining. However, the low median working experience of miners did not allow for another division as the amount of more experienced miners was very low. Therefore, the hypothesis that working as a miner for a long time period has a negative effect on HRQoL could not be confirmed.

### Strength and limitations

A number of limitations exist that have to be taken into account. Firstly, limitations result from the target and sample population and the possibility of selection bias, as the method of snowball sampling has been used. By using community leaders to get in contact with the miners, the participants of the study might only represent a specific subgroup of the miners in Kadoma, with distinctive attitudes that differ from other subgroups. Mitigation of this bias was attempted by visiting different mines and meeting places, contacting miners who were not volunteering to participate at first glance and changing the location of data collection several times a day even within one mine.

Additionally, problems with information acquisition and interviewer effects might have occurred. Not all miners were willing to talk to foreign interviewers or felt comfortable giving information about ASGM, due to the still insecure legal situation. Also, questions could be misunderstood due to language barriers and social desirability bias might have played a role. Miners maybe adapted their answers towards what they assume we wanted to hear. Taking the sensitivity of the topic into account, the study was conducted in close collaboration with partners and translators. Next to the German researcher, the research team consisted of four Zimbabweans, which helped to gain the trust of miners, translated if necessary and had important roles in the data collection. Despite inquiries were conducted outside, it was mostly avoided that bystanders could listen.

Further, it is important to ascertain if the EQ-5D questionnaire is the appropriate tool to describe the HRQoL of the Zimbabwean mining population. The other two studies we found, which have determined the QoL in mining regions, have used different questionnaires: Noronha & Nairy [41] developed their own tool, while Amponsah-Tawiah et al. [29] used Cummins seven domains of QoL [54]. Due to the difference in study design of those two studies, comparisons are difficult. The development of an own tool obviously leads to a higher adjustment to the specific situation and study design. The EQ-5D questionnaire has been originally developed to provide a simple, generic measure for clinical and economic appraisal. In recent years, it has been increasingly used in population health studies as well, but is known to be more responsive in severe conditions and can be unable to detect smaller changes in mild conditions [55]. However, the main reason we used the EQ-5D tool was to create a data set that is eligible for further comparisons and easy to apply. Further research is needed to investigate if the EQ-5D questionnaire is able to capture the HRQoL of miners appropriately.

Also, the population of Glenview was the only population with existing HU and VAS value data from Zimbabwe. The authors concluded that the sample population of the study was probably higher educated than the general urban Zimbabwean population, but that their results are credible and comparable to other studies. However, representativeness to other urban population remains questionable [37].

The effect of the healthy worker bias has already been addressed in this paper and we assume that it had considerable effect on our findings, as in fact it is not possible to work as a miner without suffering from major injuries or diseases. Unfortunately, we could not find a way to counteract this bias.

Finally, the small sample size made the statistical analysis difficult and lead to variance homogeneity. We used statistically robust tests to address this issue, but we believe this is one of the reasons that most results are statistically not significant. As the time for our field work was limited, we tried to question as many miners as possible, given the circumstances. It has to be stated, however, that this research is cross-sectional, therefore causality cannot be proven.

However, due to the paucity of research examining the HRQoL of miners, especially of small-scale miners, we believe the presented results can be useful in further decision-making processes. Also, the identified HU values can be used for comparisons over time and with other populations. As the population of artisanal and small-scale miners is difficult to access, the more knowledge is gained about their living conditions and self-perceived QoL, the better future interventions can be adapted to the miners’ health needs.

## Conclusion

In contrast to our hypothesis, the self-reported HRQoL of artisanal and small-scale miners was not significantly lower than that of the urban population. However, we believe that the reason for this is not that the numerous health hazards of ASGM have no impact on HRQoL, but because the urban Zimbabwean population faces many problems as well that negatively affect HRQoL. Education was identified as being very important for higher HU values and HRQoL, which can build a basis for future interventions. However, as remaining subgroup analyses were not significant, further research is needed to investigate if the small sample size played a role and which factors have the biggest impact on HRQoL of miners.

In total, many miners reported health problems in the cognition dimension, confirming the choice of including this additional dimension into the study and proving that this dimension is of high relevance for the miners’ health and HRQoL. Additionally, this dimension seem to play an important role for the differences of HRQoL between miners with or without mercury contact, which is relevant in context of the *Minamata Convention on Mercury*.

The findings of this study can be used for further comparisons between different populations and can build a basis for future investigations of HRQoL of small-scale gold miners.

## Supplementary information

**Additional file 1.**

## Data Availability

The datasets used and/or analysed during the current study are available from the corresponding author on reasonable request.

## Abbreviations

ASGM: Artisanal and small-scale gold mining
HRQoL: Health related quality of life
EQ-5D-3L+C: Five EuroQol dimensions accompanied by the cognition add-on questionnaire
VAS: Visual analogue scale
HU: Health utility
ASM: Artisanal and small-scale mining
GDP: Gross domestic product
TTO: Time trade-off
QoL: Quality of Life
