# Supplementary material for: Comparing the self-reported health-related quality of life (HRQoL) of artisanal and small-scale gold miners and the urban population in Zimbabwe using the EuroQol (EQ-5D-3L+C) questionnaire: a cross-sectional study
Source: Health Qual Life Outcomes. 2020 Jul 29;18:253. doi: 10.1186/s12955-020-01475-0 (PMC7390189; doi:10.1186/s12955-020-01475-0)
Supplement: Supplementary file 1 — Additional file 1. [file 12955_2020_1475_MOESM1_ESM.docx]

1. Online Supplementary

**
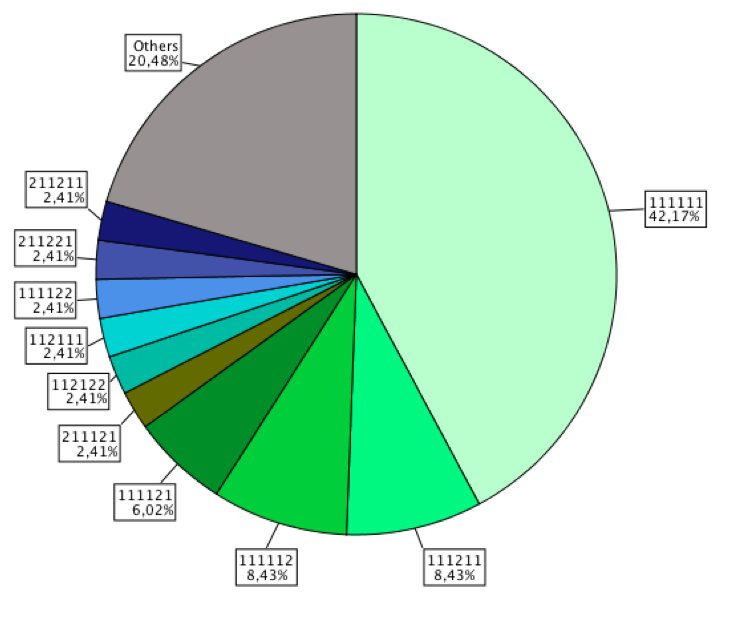
**

Online Supplementary Figure 1: Prevalence of the 10 most frequent EQ-5D+C health states in the sample. Others refer to further reported health stated (no missing values exist).

Online Supplementary Table 1: Demographic details of study sample compared to population figures.

|  | **SAMPLE FREQUENCY** | **SAMPLE PROPORTION %** | **POPULATION PROPORTION %*** |
| --- | --- | --- | --- |
| **Gender** |  |  |  |
| Males | 69 | 83.1 | 48.1 |
| Females | 14 | 16.9 | 51.9 |
| Total | 83 |  |  |
| **Age category** |  |  |  |
| 15-24 | 8 | 9.6 | 20.0 |
| 25-34 | 41 | 49.4 | 15.7 |
| 35-44 | 21 | 25.3 | 9.7 |
| 45+ | 13 | 15.7 | 13.3 |
| Total | 83 |  |  |
| **Highest Education** |  |  |  |
| Primary | 21 | 25.3 | 47.8 |
| Secondary | 57 | 68.7 | 36.1 |
| Post School | 5 | 4.8 | 7.5 |
| Total | 83 |  |  |
| **Income** |  |  |  |
| < 100$ | 10 | 12.0 |  |
| 100 – 500$ | 40 | 48.2 |  |
| 501 – 1000 $ | 15 | 18.1 |  |
| 1001$ + | 13 | 15.7 |  |
| Total | 78 |  |  |
| Missing | 5 |  |  |
| **Years in Mining** |  |  |  |
| < 1 | 12 | 14.5 |  |
| 1 – 5 | 33 | 39.8 |  |
| 6 – 10 | 17 | 20.5 |  |
| > 10 | 21 | 25.3 |  |
| Total | 83 |  |  |

Online Supplementary Table 2: Overview of all reported health states by miners (n=83).

| **Health States** | **Frequency** |
| --- | --- |
| 111111 | 35 |
| 111112 | 7 |
| 111211 | 7 |
| 111121 | 5 |
| 111122 | 2 |
| 112111 | 2 |
| 112122 | 2 |
| 211121 | 2 |
| 211211 | 2 |
| 211221 | 2 |
| 111113 | 1 |
| 111123 | 1 |
| 111132 | 1 |
| 111212 | 1 |
| 111311 | 1 |
| 112112 | 1 |
| 112113 | 1 |
| 112211 | 1 |
| 121131 | 1 |
| 121132 | 1 |
| 121212 | 1 |
| 122221 | 1 |
| 131113 | 1 |
| 211113 | 1 |
| 212222 | 1 |
| 212322 | 1 |
| 221113 | 1 |
| Total | 83 |

Online Supplementary Table 3: Health states of miners who worked with mercury (n=63).

|  | **Health State** | **Frequency** | **Percent** |
| --- | --- | --- | --- |
| 1 | 111111 | 24 | 38,1 |
| 2 | 111112 | 6 | 9,5 |
| 3 | 111211 | 6 | 9,5 |
| 4 | 111121 | 3 | 4,8 |
| 5 | 111122 | 2 | 3,2 |
| 6 | 112111 | 2 | 3,2 |
| 7 | 112122 | 2 | 3,2 |
| 8 | 211121 | 2 | 3,2 |
| 9 | 211211 | 2 | 3,2 |
| 10 | 111113 | 1 | 1,6 |
| 11 | 111123 | 1 | 1,6 |
| 12 | 111212 | 1 | 1,6 |
| 13 | 111311 | 1 | 1,6 |
| 14 | 112112 | 1 | 1,6 |
| 15 | 112211 | 1 | 1,6 |
| 16 | 121131 | 1 | 1,6 |
| 17 | 121132 | 1 | 1,6 |
| 18 | 121212 | 1 | 1,6 |
| 19 | 122221 | 1 | 1,6 |
| 20 | 131113 | 1 | 1,6 |
| 21 | 211113 | 1 | 1,6 |
| 22 | 211221 | 1 | 1,6 |
| 23 | 212322 | 1 | 1,6 |
|  | **Total** | **63** | **100,0** |
